# Perspective: A toolbox for protein structure determination in physiological environment through oriented, 2D ordered, site specific immobilization

**DOI:** 10.1063/1.4981224

**Published:** 2017-04-14

**Authors:** M. Altissimo, M. Kiskinova, R. Mincigrucci, L. Vaccari, C. Guarnaccia, C. Masciovecchio

**Affiliations:** 1 Elettra Sincrotrone Trieste, S. S. 14 km 163, 34149 Trieste, Basovizza, Italy; 2 International Centre for Genetic Engineering and Biotechnology, Padriciano 99, 34149 Trieste, Italy

## Abstract

Revealing the structure of complex biological macromolecules, such as proteins, is an essential step for understanding the chemical mechanisms that determine the diversity of their functions. Synchrotron based X-ray crystallography and cryo-electron microscopy have made major contributions in determining thousands of protein structures even from micro-sized crystals. They suffer from some limitations that have not been overcome, such as radiation damage, the natural inability to crystallize a number of proteins, and experimental conditions for structure determination that are incompatible with the physiological environment. Today, the ultra-short and ultra-bright pulses of X-ray free-electron lasers have made attainable the dream to determine protein structures before radiation damage starts to destroy the samples. However, the signal-to-noise ratio remains a great challenge to obtain usable diffraction patterns from a single protein molecule. With the perspective to overcome these challenges, we describe here a new methodology that has the potential to overcome the signal-to-noise-ratio and protein crystallization limits. Using a multidisciplinary approach, we propose to create ordered, two dimensional protein arrays with defined orientation attached on a self-assembled-monolayer. We develop a literature-based flexible toolbox capable of assembling different kinds of proteins on a functionalized surface and consider using a graphene cover layer that will allow performing experiments with proteins in physiological conditions.

## I. INTRODUCTION

The determination of the structure of proteins and other macromolecules has historically been prerogative of X-ray crystallography. One of the technique's requirements is the growth of high-quality crystals, which need to be sufficiently large to efficiently diffract X-rays while withstanding radiation damage. This method suffers from two noteworthy constraints, which make structure determination extremely difficult or sometimes impossible. First, many bio-molecules cannot be crystallized, while the production of large, high quality crystals is not possible with many others. These restrictions are most severe for large protein complexes, such as membrane proteins, which participate in a range of biological processes and also are interesting pharmaceutical targets for future drugs. The second limitation is the unavoidable X-ray radiation damage. The crystal size and radiation damage are inherently linked since reducing the dose delivered to a single molecule requires large crystals amplifying the signal through Bragg diffraction. Therefore, synchrotron-based experiments are usually performed with cryo-cooled crystals in order to reduce the mass-transport rate due to radiation damage.

One of the breakthroughs using the ultrafast and coherent nature of intense X-ray pulses generated by a free-electron laser (FEL) is single-shot, coherent diffractive imaging performed before the destruction of the object, with diffraction-limited resolution. In life sciences, the ultimate goal for FELs is to solve the structure of large biomolecules without crystallization, and this ambitious goal calls for the development of new experimental methodologies.^1,2^ The first attempts to solve the structure of large biomolecules include the development of serial femtosecond crystallography (SFC) at room temperature, combined with the use of jets for the continuous delivery of micro-/nano-crystals into the FEL hit zone. The structure is subsequently determined from collecting many thousands of diffraction patterns.^3–5^ Time-resolved SFC experiments highlighted photo-induced changes in the electronic structure due to charge transfer, encoded in the diffraction pattern of the microcrystals under examination.^6,7^ Interestingly, serial nano-crystallography has also become feasible with focused beams at synchrotrons,^8^ while microfluidic devices for protein crystallization and on-chip diffraction studies have been developed and demonstrated.^9^ One of the main limitations for a broad applicability of SFC is that thousands of crystals are needed to obtain complete datasets. Considering that only a relatively small fraction of successful hits generates useful diffraction patterns, in order to become a routine analytical tool, SFC still lacks an easy, reliable, and inexpensive way of producing a large number of nanocrystals.

The limited access to perfect crystals has been an unsolved problem in protein crystallography for decades, further adding to the difficulty in solving the structure of complex bio-molecules. It is interesting to note that some recent diffraction studies performed on X-ray free-electron lasers (XFELs) have demonstrated that an imperfect crystal structure is not a constraint. The “neglected” weak continuous scattering arising when the crystals become disordered contains key information to overcome the resolution limits and to solve the tricky phase puzzle.^10^ As shown in this paper, the continuously modulated background fully encodes the waves diffracted from individual single molecules. Thus, by using coherent diffraction imaging methods, it is possible to obtain real-space images of macromolecules with a higher lateral resolution than that is obtainable by ordinary Bragg diffraction.

A common feature of all the achievements summarized above is that all the protein structures solved at synchrotron or FEL sources are based on the use of 3D crystals. It is interesting to note how, even in the case of “in-vivo” room temperature SFC experiments, the protein crystal does not represent the protein in its more natural arrangement. Until now, 2D-crystallography has almost exclusively been the area of transmission electron microscopy (TEM). Significant progress in protein structure determination and lipid interaction has been made thanks to the use of cryo-TEM and the development of algorithms for recovering amplitudes and phase information from the recorded TEM images.^11,12^ Given the reduced amount and fixed-target sample delivery in a near-native environment, single-shot 2D protein crystallography with FELs was suggested as an attractive alternative^13,14^ and has been recently demonstrated by proof-of-principle experiments at Linac Coherent Light Source. One can also argue that the 2D approach adds the ability to explore the protein function and dynamics and is also an intermediate step towards the extremely ambitious XFEL case—atomic imaging of individual bio-molecules.

As in the 3D case, sample preparation is a crucial step for the 2D case, where fixed-target solutions have to be developed. For 2D cryo-TEM studies, most often the protein crystals are grown embedded within a lipid bilayer, whereas the first XFEL experiments used dices of SiN windows for harvesting 2D crystals, which are then covered with a thin C film. These first experiments indicated that in order to overcome the present resolution limits of 7 Å and truly exploit the unique XFEL properties, an improvement of both sample preparation and data analysis is of the essence.

In this perspective, we put forward a new sample delivery method based on fabricating sample supports for hosting the target protein in a near-native environment. Our approach makes use of patterned silicon nitride membranes, over which a graphene cover ensures the stability of the liquid layer hosting the protein of interest (POI). This is in turn covalently bound onto a chemically modified surface, so that an ordered array is produced in aqueous solution, and kept in an environment as close as possible to physiological conditions during data acquisition, a condition that is not compatible with standard protein diffraction methods. The chemical binding method of the POI to the surface must ensure that the layer is 2D-ordered and the protein is in its biologically active state. Further, we present an experimental approach based on free electron laser diffraction before destruction, with the final aim of our work being the determination of the protein structure in physiological conditions.

## II. DISCUSSION

### A. Directed protein immobilization

The immobilization of a POI on a surface can be accomplished through several methods, both physical and chemical. Although physical methods are in general easier and more straightforward to develop, they tend to yield a disordered array of the POI on the substrate of choice. The added constraint of having an ordered array severely restricts the available choices. The binding process should satisfy the following criteria:

(1) It covalently binds two unique and mutually reactive groups, one on the protein and the other on the surface;
(2) It is bio-orthogonal, i.e., it should not appear in, or cross-react with, any of the groups of endogenous amino acids;
(3) It works efficiently under physiological conditions and without the use of harsh chemicals that could cause the denaturation of the POI;
(4) It does not alter substantially the structure of the POI, so as to leave it in its functional state;

The added complication is that the structure of each protein varies hugely; this impacts directly the fact that there is no single method that can be used universally to attach proteins to a surface. Thus, given the POI, the binding methodology should be chosen among a set of several ones; this set therefore constitutes a “toolbox.” A corollary of points (1) and (4) is that regardless of the chemistry used to bind the POI to a surface, the reacting moiety should be small in comparison to the POI. Engineering the position of proteins on a surface in ordered arrays has been studied extensively by the protein biochip community.^15,16^ The POI will have to be modified with a unique chemical group or sequence at a site-specific location in order to ensure a covalent and oriented binding to the surface, which in turn must also be adapted to “receive” the POI. The adaptation of the POI can be accomplished through its recombinant expression by a suitably genetically modified vector. Several classes of chemical reactions can be employed to engineer proteins onto a surface and are reviewed in a number of papers.^15–19^ Six of those classes are particularly suited for the purpose of anchoring: catalyzed cyclo-addition,^20,21^ modified Staudinger ligation,^22,23^ Diels-Alder cyclo-addition,^24,25^ thiol-ene additions,^26,27^ oxime ligation,^28,29^ and Expressed Protein ligation, such as Split-intein-mediated ligation^30,31^ or farnesyltransferase-related methods.^32^ While these are not strictly single chemical reactions, they are used to bind a POI to a surface by means of the naturally occurring protein trans-splicing process^33^ (split-intein mediated case) or farnesyltransferase mediated binding.^19^

All these reactions satisfy the 4 requirements listed above, and therefore, they constitute an appropriate “toolbox” for anchoring ordered 2D arrays of proteins on a substrate. Their effectiveness has already been reported.^34^

Among the methods described above, we will concentrate on the last since it is able to yield a traceless attachment of the POI to a gold surface, with minimal POI modification. The technique has been first described in Ref. 30. The basic idea described there is to genetically engineer a suitable expression vector, with the most common hosts being *Escherichia coli, Pichia pastoris,* or *Saccharomyces cerevisiae,* to express the POI with an N-intein fragment attached to its C-terminal. On the surface, the complementary C-intein fragment is attached to a modified polyethylene glycol (PEG) linker. When the C-intein and the N-intein fragments interact, they form an active intein domain, binding the POI to the surface. The split intein is naturally spliced into the solution, leaving the POI attached to the surface. Generally speaking, a self-assembled monolayer (SAM) of two differently modified PEG chains is used, given that they are readily available and chemically well understood.^35^ The shorter of the two is used as a spacer, separating the substrate from the solution, while the second longer one is modified to bind with the POI.

It is worthwhile noting that a different ordering strategy is required in order to solve the structure of membrane proteins in their native environment. These constitute 20%–30% of the total proteome but represent about 1% of determined protein structures, due to difficulties in their crystallization. Cryo-TEM has been one of the main tools of choice for integral membrane protein (IMP) structure reconstruction. The base of the 2D crystallization work behind cryo-TEM IMP structure reconstruction is a work recognizing as early as 1992 (Ref. 36) that IMP can form 2D crystals when inserted into an artificial phospholipid bilayer.^37,38^ Due to the nature of IMPs, ad-hoc crystallization conditions have to be determined for each protein.^39–41^ A toolbox for the 2D crystallization of IMPs is therefore already well established, and we suggest that FEL-based in particular, and more in general X-ray-based, IMP structure reconstruction should follow the methodologies found in the literature.

### B. Proposed methodology

**FIG. 1.**
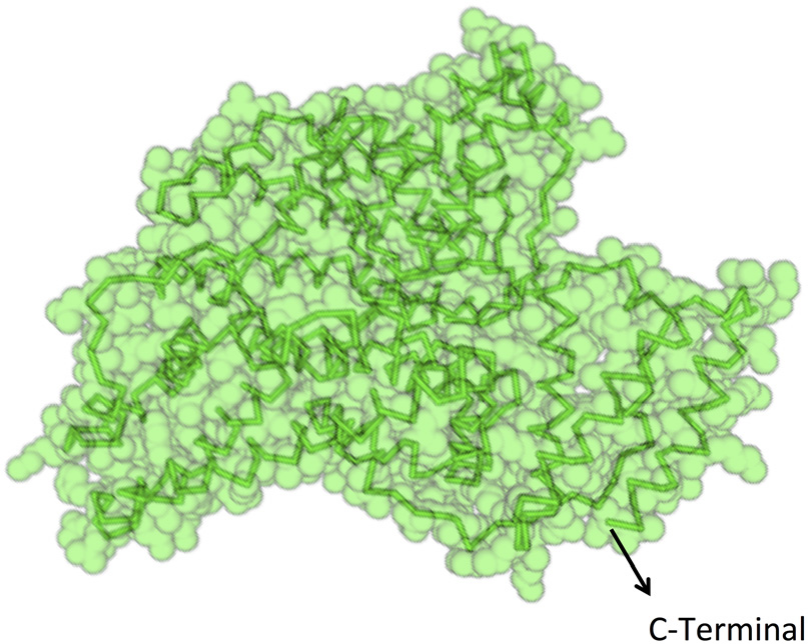
Human Serum Albumin. The backbone of the protein is shown superimposed to a space fill model. The C-terminal of the protein is also indicated. Data are taken from Refs. 42 and 43.

**FIG. 2.**
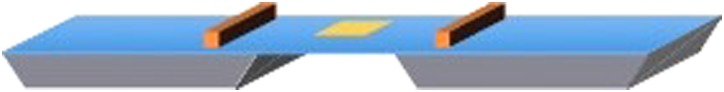
Schematics of a single FEL-transparent area. The SiN membrane (in light blue) is used as a carrier for the 10 *μ*m wide, 10 nm thick Au square (in gold). Outside of the membrane perimeter, the area is corralled by the patterned photoresist, used as a prop for the graphene cover (not shown in the figure) to keep the sample area wet and separated from each other.

The tight integration of the sample delivery system and POI orientation will maximize the chance of a successful protein structure reconstruction. At first, we focus on Human Serum Albumin (HSA), in order to show how the proposed method would allow investigating the structure of a well-characterized system in a physiological environment. The HSA structure has been resolved in 1999 by Sugio and coworkers;^42^ all the relevant protein data can be found at the Protein Data Bank.^43^ HSA has an exposed C-terminal that allows for an N-intein group to be attached through recombinant techniques, see Figure 1. Further, being a protein found in blood, it is safe to assume that the protein will be in its functional conformation in a physiological solution. The protein will be bound on Au, through a tailored SAM. Standard photolithography techniques will be used to open spatially separated SiN membranes and define 10 nm thick Au, 10 *μ*m in side, square patches, using 2 nm Ti as an adhesion layer. Care must be taken in order to minimize the rms roughness of the Au layer, as this would impact directly the relative positions of the POI's atoms. The typical rms roughness value obtained is around 2 Å. An optical resist will be patterned via photolithography in order to “corral” each of the membranes and serve at the same time as the support for the cover. Graphene is the cover's ideal material,^44^ due to its very low absorption. The photoresist props also serve to keep the POI environment wet and to create separate micro-environments, in order to minimize the possibility of water leakage after each FEL shot. Figure 2 schematizes the end result, excluding the graphene cover. As an alternative, a second nitride membrane could be used for sealing the wet chamber.

To define the SAM, we will proceed as described in Ref. 19 with a slight modification. A dithiocarbamate (DTC)-modified polyethylene glycol (PEG) will serve as a linker, with the loose end (i.e., the one not attached to the underlying gold layer) modified by a C-intein domain as described in Refs. 16 and 30. A shorter, unmodified DTC-PEG layer will be used as a spacer for the anchoring points. The density of the anchoring points can be controlled by simply adjusting the relative concentration of the 2 DTCs in solution. The POI (HSA) will be recombinantly over-expressed by genetically engineered Pichia Pastoris^45^ or by other suitable eukaryotic vectors. One of the appeals of the binding process proposed here is that the POI could bind to the surface directly from the cell culture medium (if the vector expresses the POI in that way), thus avoiding lengthy purification steps. The POI will be covalently linked to the Au surface via the intein-mediated trans-splicing process as described above, while all the other cell debris will be washed away with an appropriate rinsing step.

**FIG. 3.**
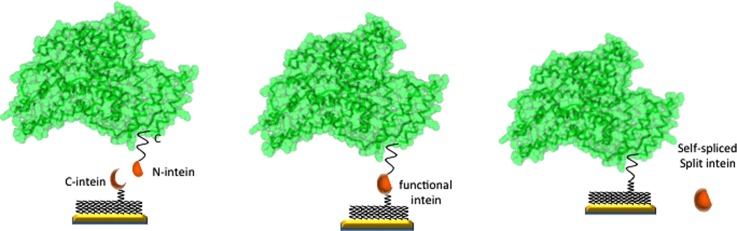
Schematics of the HSA covalent binding process to the surface.

Figure 3 shows schematically the binding process. By rough calculations, according to the data published on the Protein Data Bank, we estimate that there will be in excess 2.5 × 10^6^ HSA proteins per 10 *μ*m square Au patch. What we believe is particularly interesting about our methodology is the fact that any protein of known sequence can be recombinantly modified to incorporate into either its N- or its C-terminal a suitable moiety that will bind to a chemically complementary substrate, regardless of the ability of the POI to crystallize. Together with a uniquely defined surface anchoring point, the protein-protein interactions due to charge distribution and steric hindrance will be in accordance with the POI onto the chemically engineered surface. This has been demonstrated in the literature by, for example, Refs. 19 and 46.

### C. Experimental needs

In this section, we will discuss the experimental requirements needed to reconstruct the protein structure. To avoid sample degradation due to radiation damage, FEL pulses represent a valuable choice.^47^ According to Ref. 48, diffraction patterns can be easily acquired to better than 8.5 Å resolution, from two-dimensional protein crystal samples less than 10 nm thick and at room temperature. While this achievement represents a big step in the direction of determining the protein structure in the physiological condition, the limits of Ref. 48 were basically related to the fact that the protein had random orientation on the substrate. As the authors pointed out: “The extent to which multiple lattice or powder diffraction XFEL data can be reliably used for structure determination from 2D crystals is currently unclear.” Using our approach in the case of HSA, we can align up to about 2.5 × 10^8^ proteins on a SiN substrate as large as 100 *μ*m^2^.

**FIG. 4.**
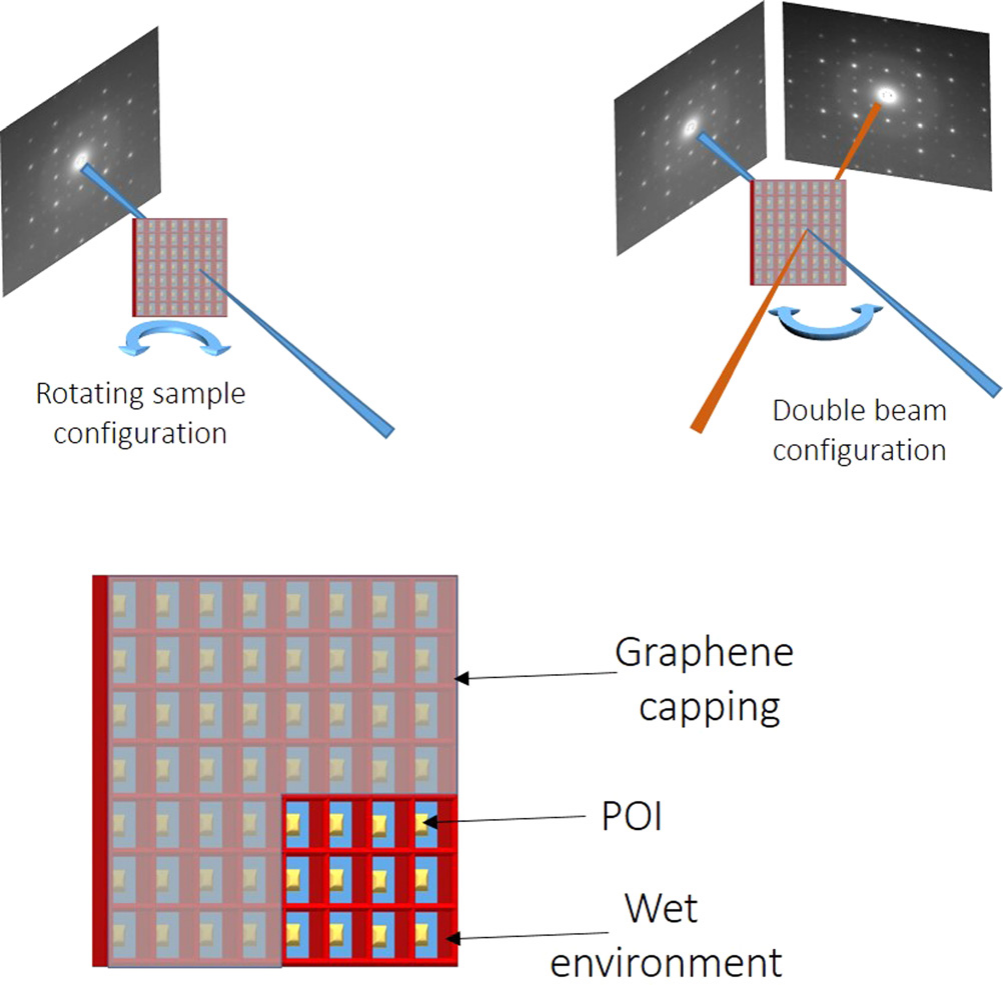
Proposed set-up for the single beam diffraction (top left) and for double beam diffraction (top right). The angle of incidence of the beam with respect to the surface and/or the inter-beam angle can be varied to obtain complementary diffraction patterns.

This will strongly increase the intensity from Bragg diffraction and, moreover, the use of a large focal spot will prevent electron stripping from the atoms upon pulse arrival, thus decreasing the scattering from unbound electrons and further increasing the signal-to-noise ratio. Finally, the use of a graphene cover allows keeping the protein in the physiological condition. Focal spot sizes can be easily varied from 10 *μ*m to 1 mm using standard optics set-ups, thus defining the maximum POI island size that can be illuminated by a single shot. In the case of ablation, samples can be raster scanned. The diffraction pattern of each POI patch may be acquired using a suitable area detector. When single shot techniques have been applied to nano-crystals, the 3D structure of the protein has been recovered by sorting and merging diffraction patterns obtained by randomly orienting the crystals in jets.^3^ Using our approach, one can rotate the sample under the beam so as to precisely define the angle under which the POI's diffraction pattern is recorded. Alternatively, and with the same goal, a set-up consisting of two crossed FEL beams^49^ (Figure 4) and/or two detectors may be employed to simultaneously record the diffraction pattern of the same area from two different directions.

**FIG. 5.**
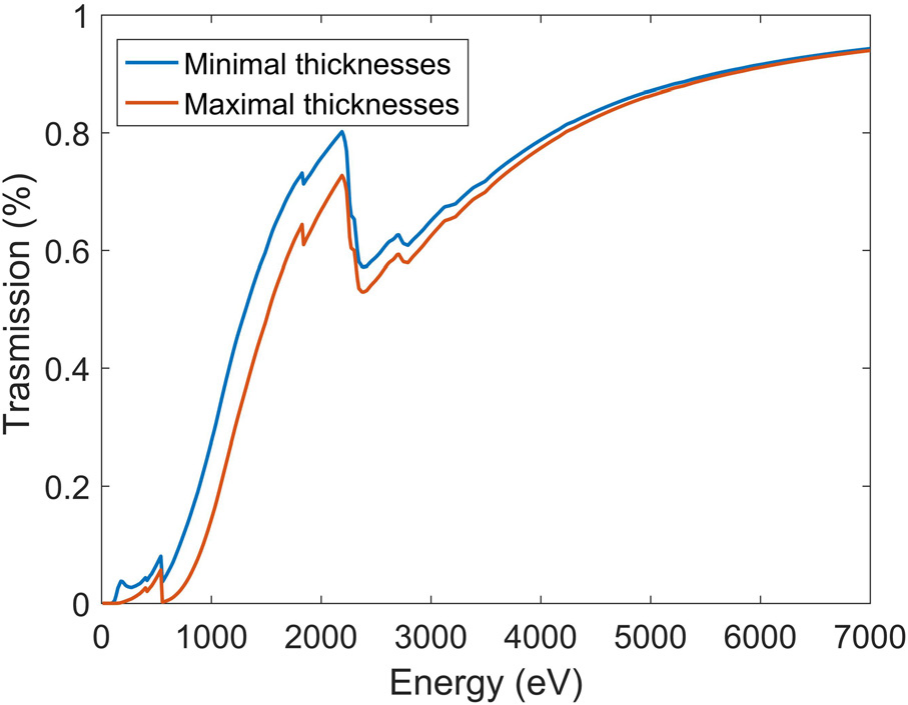
Estimated sample transmission for 50 nm of Silicon Nitride and 0.5 *μ*m of water (blue curve) and for 100 nm of Silicon Nitride and 2 *μ*m of water (orange curve).

Figure 5 shows the calculated theoretical transmission of the sample stack. The blue and orange curves refer to 50 nm SiN/0.5 *μ*m water and 100 nm SiN/1 *μ*m water, respectively. For both the curves, a 2 nm Ti and 10 nm Au bilayer has been added. As expected, a monolayer of proteins does not contribute substantially to the total absorption of the stack. The proposed structure has a static transmission value around 40% in the least favorable case for photon energies above 1 keV.

In order to calculate the signal improvement, it is interesting to estimate the increase in the Bragg signal due to the number of proteins aligned on the surface and under the beam. As in any interference problem, the strength of the main peak is proportional to the number of diffracting objects squared,^50^ while the ratio between different order/directions is governed by more complex relations.^51^ For photosystem I as studied by Ref. 3 using single FEL shot methods, an intensity in the Bragg spots ranging from 5 × 10^3^–5 photons was recorded as a function of angle. The lowest reported crystal size (P6_3_ crystal structure with a diameter of 160 nm and a diameter to length ratio of 1:2) corresponds to a number of proteins ranging from 270 to 540. With our sample delivery method used with the same photosystem I, we expect to obtain a Bragg peak intensity which is at least a factor 6 × 10^6^ higher since a POI SAM of 100 × 100 *μ*m should contain ∼1.3 × 10^7^ proteins. Similar ratios are thus expected for other proteins.

The recent development of Free Electron Laser Facilities has opened up different schemes for the production of photon pulses.^52^ One recently highlighted possibility is to allow only a single superradiant spike of the electron bunch self-emission to be amplified in an exotic configuration. This mode may produce pulses as short as 500 as, with the drawback of a limited number of photons per pulse. Using the parameters of the European XFEL under commissioning in Hamburg,^53^ the expected flux would be about 10^10^ photon per pulse. It has been demonstrated that FEL single molecule imaging will strongly benefit from using sub-femtosecond pulses, due to the significant reduction in radiation damage and the formation of preferred multisoliton clusters, which reshape the overall electronic density of the molecular system when using longer pulses.^54^ While the expected number of photons per pulse in the superradiance scheme would be too low to get the diffraction pattern from a single molecule, this is not the case for the 2D array sample delivery method we introduce here, thanks to the several orders of magnitude enhancement of the scattering power due to the large number of molecules involved in the process.

## III. CONCLUSIONS

In this article, we proposed a new method capable of delivering two-dimensional, aligned arrays of bio-molecules. The alignment should increase the signal-to-noise ratio by several orders of magnitude in FEL-based diffraction experiments, as compared with other reported studies. The sample will be a two-dimensional crystal containing up to billions of ordered macromolecules that will be kept in a near-native environment. The perspective route presented above follows a multidisciplinary approach based on the existing literature. We discussed in detail the advantages of this methodology that we highlight in the following:

(1) It will permit us to reveal the structure of macromolecules in general, and proteins more in particular, that cannot be crystallized and, therefore, cannot be studied by classical crystallography;
(2) The 2D protein array would allow using FEL pulses produced in exotic configurations with a shorter pulse duration (namely few attoseconds). This would definitively avoid sample radiation damage occurring during the diffraction process.
(3) The measurements can be carried out in physiological conditions;
(4) Only a moderate sample quantity will be necessary. This is extremely important when samples are difficult or very expensive to produce.

We note finally that the sample delivery method we propose here also allows for pump-and-probe experiments, whereby a visible laser excites the POI, and the FEL beam probes its structure post-excitation. This could, for example, enable us to determine the dynamic conformational changes in the structure of a protein of known structure (i.e., a photosynthetic centre) before, during, and after its excitation by visible-light.
